# Positive aspects of caregiving among informal caregivers of persons with dementia in the Asian context: a qualitative study

**DOI:** 10.1186/s12877-023-03767-8

**Published:** 2023-01-27

**Authors:** Qi Yuan, Yunjue Zhang, Ellaisha Samari, Anitha Jeyagurunathan, Richard Goveas, Li Ling Ng, Mythily Subramaniam

**Affiliations:** 1 Research Division, Institute of Mental Health, Buangkok Green Medical Park, 10 Buangkok View, Singapore, 539747 Singapore; 2 Department of Geriatric Psychiatry, Institute of Mental Health, Singapore, Singapore; 3 Department of Psychological Medicine, Changi General Hospital, Singapore, Singapore

**Keywords:** Informal caregiving, Dementia, Positive aspects of caregiving, Qualitative methodology, Asian

## Abstract

**Background:**

Positive aspects of caregiving are important coping resources for informal caregivers of persons with dementia (PWD). However, existing studies mostly focused on caregivers from western societies and less attention was paid to the potential cultural differences. This study aims to explore positive aspects of caregiving in the Asian context.

**Methods:**

A qualitative methodology with semi-structured interviews was adopted. A total of 29 informal caregivers of PWD in Singapore were interviewed from Apr 2019 to Dec 2020. All the interviews were audio-recorded and transcribed verbatim for the analysis. Inductive thematic analysis was conducted.

**Results:**

The results revealed a total of three major themes with 11 sub-themes including: 1) positive aspects within self (i.e., better understanding of dementia and caregiving, personal growth, role satisfaction, and improved awareness of self-care); 2) positive aspects between caregiver and PWD (i.e., chance to demonstrate filial piety towards PWD, happiness and positive attitudes of PWD, positive interactions with PWD, and closer relationships with PWD); and 3) positive aspects between caregiver and others (i.e., empathy towards other caregivers, befriending peers, and sharing dementia and caregiving knowledge with others).

**Discussion:**

Findings from this study improved our understanding on positive aspects of caregiving among informal caregivers of PWD in the Asian context. In addition to similar themes across cultures such as personal growth, our study identified a few unique themes like improved awareness of self-care and chances to demonstrate filial piety. For future studies targeting Asian caregivers, it is necessary to include these cultural-specific positive aspects of caregiving.

**Supplementary Information:**

The online version contains supplementary material available at 10.1186/s12877-023-03767-8.

## Background

Dementia is a condition that is prevalent among older adults. According to the World Health Organization, there are around 55 million people living with dementia globally, and this number is expected to increase further due to population aging [1]. Dementia impacts individuals in different ways, such as affecting their cognitive function (e.g., memory loss or confusion), behaviour (e.g., wandering and asking repeated questions), and psychological behaviour (e.g., agitation, aggression, depression or delusions) among PWD [2]. Due to the progressive deteriorations, they lose their ability to carry out activities of daily living gradually, become more dependent, and require care and assistance from others. Assistance such as helping PWD with their activities of daily living, engaging PWD in different activities, accompanying PWD to hospital appointments and financially supporting them, are mostly provided by informal caregivers like family members of PWD [3]. However, the caregiving experience is not an easy one. According to previous studies, caregivers often face a lot of challenges including behavioral and psychological problems of PWD [4], financial strain [5], high workload from assisting in the activities of daily living of PWD [6], and social isolation from long hours of caregiving [7]. These stressors can lead to negative consequences such as high caregiving burden [4] or potential depression [8, 9] among caregivers.

However, apart from the above mentioned challenges, informal caregivers of PWD also encounter positive experiences while taking care of their loved ones [10], and these positive aspects can play an important role in the process of caregiver stress [11] and well-being [10]. According to the transactional stress and coping model, stress occurs when individuals appraise the environment as significantly impairing their well-being and exceeding the available coping resources [12]. Positive aspects of caregiving are one of the coping resources that can buffer the impacts of caregiving stressors [13]. For example, in a previous study among dementia caregivers, a significant negative relationship between positive aspects of caregiving and caregiver burden was reported [14]. Another longitudinal study also reported a positive relationship between positive aspects of caregiving with caregiver well-being and satisfaction of life [15]. In addition, positive aspects of caregiving might also affect caregiver outcomes indirectly through other factors such as self-efficacy. More specifically, studies suggested that self-efficacy is a major component of positive aspects of caregiving [16] and it is one of the resilience factors for caregivers’ health outcomes [17].

There are different categories of positive aspects of caregiving among informal caregivers of PWD. A literature review of qualitative studies exploring positive aspects of caregiving identified a few key categories including role satisfaction, emotional rewards, personal growth, competence and mastery, relationship gains, sense of duty, and reciprocity [18]. However, among the 14 studies included in this review, 13 of them were on western caregivers [18] and only one was on caregivers from Asia (i.e., Singapore specifically) [19]. In a more recent review of positive aspects of caregiving, four main domains were identified: a sense of personal accomplishment and gratification, feelings of mutuality in a dyadic relationship, an increase of family cohesion and functionality, and a sense of personal growth and purpose in life [13]. But this review also highlighted the same concern, i.e., the lack of studies on positive aspects of caregiving among Asian caregivers [13].

Research revealed that there are cultural differences between western and Asian caregivers. More specifically, the Confucian value of filial piety which leads to the belief that it is one’s responsibility to take care of family members is prevalent among Asian caregivers [20], especially among Chinese caregivers [21]. A previous review suggested that filial piety could affect caregivers’ attitudes towards help seeking and other caregiving processes such as appraisal of stress, coping and social support [22]. In another study, filial piety was found to partially mediate the relationship between the behavioral and psychological symptoms of PWD and positive aspects of caregiving among Chinese dementia caregivers [23]. These cultural values might affect how caregivers appraise their caregiving experiences, in the sense that if the caregiver has such a belief, they are more likely to view the caregiving experience more positively [23]. However, this has seldom been discussed in the other qualitative studies on positive aspects of caregiving done in Asia [19, 24].

### Study aims

The current study adopted a qualitative approach to explore positive aspects of caregiving among informal caregivers of PWD in Singapore to understand the concept in an Asian context.

## Methods

### Study design

This study was part of a larger qualitative project which aimed to understand the caregiving experiences of informal caregivers of PWD (i.e., both caregiving challenges and positive caregiving experiences) in Singapore. An interpretive approach was adopted. Data was collected from Apr 2019 to Dec 2020. The Standards for Reporting Qualitative Research (SRQR) checklist was used to guide the reporting of the study [25].

### Participants

The participants were mainly recruited via referrals from the clinicians who were treating their relative or friend with dementia in the outpatient and satellite clinics of Institute of Mental Health (the only tertiary mental health service provider in Singapore) and a geriatric clinic in Changi General Hospital, Singapore. In order to maximize the recruitment efforts, the study team also approached caregivers who had participated in a prior study and had given permission to be contacted for future studies. Snowball recruitment was also used by encouraginge participants to refer their peers to join the study. Caregivers needed to meet the following criteria to be eligible for the study: 1) Singapore residents (including citizen and permanent residents); 2) aged 21 years and above; 3) self-identified as the key caregiver of an individual who has been formally diagnosed with dementia; 4) and able to communicate in either English, Mandarin, Malay or Tamil. Caregivers who did not visit the PWD on a weekly basis, or whose PWD were institutionalized in nursing homes at the point of recruitment, or had difficulties in understanding the consent process were excluded. The final sample consisted of 29 participants, as the researchers concluded that data saturation had been achieved – no new information to form distinct themes was observed and collected.

### Data collection

Potential participants were approached first via phone calls to check their willingness to join the study and their eligibility. Written informed consent was then obtained from all participants. After that, participants were required to fill in a short form survey on their sociodemographic information. Qualitative data were collected via semi-structured in-depth interviews. An interview guide was developed by the research team based on the literature as well as their experiences of working with local informal caregivers of PWD. Each interview was conducted by two team members who were trained in qualitative research (QY, YJZ, ES and AJ) – one interviewer and one note taker. The interviews were mainly conducted in English, with two were done in Chinese (by QY and YJZ) as preferred by participants who were more comfortable conversing in their mother tongue. The interviews were either conducted face-to-face in a private place preferred by the caregivers or online via zoom (a total of six interviews due to the COVID-19 restrictions implemented during the study period). The interviews lasted 60 min on average and ranged between 33 min and 1 h 35 min. Questions in the interviews covered topics including the reason for seeking help for the PWD, diagnosis and treatment seeking process and their feelings, daily caregiving experiences, and challenges and positive experiences encountered while taking care of the PWD. Probing questions were asked to clarify doubts and obtain richer information.

### Ethical consideration

Ethical approval was obtained from the National Healthcare Group Domain Specific Review Board in Singapore (reference number: 2018/01069).

### Analysis

All the interviews were audio-recorded and transcribed verbatim, and were checked by the study team first to ensure consistency before the analysis. An inductive thematic approach was used in the analysis [26]. More specifically, four random transcripts were selected first and were then distributed to four team members (QY, YJZ, ES and AJ). Each repeatedly reviewed the assigned transcript and worked out their own codes. Discussions were held to standardize, condense and group these preliminary codes into a codebook with clear definitions of the codes, the inclusion and exclusion criteria, as well as a few typical examples for reference. After finalizing the codebook, all four team members coded three of the same transcripts to establish the inter-rater reliability. Upon achieving a satisfactory kappa coefficient of 0.803, all the 29 transcripts were distributed to QY, ZYJ and ES for independent coding. After the independent coding process was completed, the team members had another series of discussions to review and finalize all the codes. After this step, the study team held a series of discussions to collate the codes featuring similar contents into the initial themes. These initial themes were about the most prevalent patterns emerging from the data on the research questions. Subsequently, we reviewed, modified and developed the themes (e.g., combing initial themes when there was an overlap and creating parent themes where we felt some themes while different, contributed to an overall theme – which were labelled as subthemes). Lastly, we further reviewed and refined these themes to ensure their uniqueness and defined their connections with other themes. All the coding, analysis and inter-rater tests were conducted via NVivo 11 [27]. We have included an example of the analysis, from meaning units, code, subthemes to major themes, in the supplementary table. Minimal corrections have been made to the quotes presented in this study to ensure readability. 

We have assessed the rigor of the analysis as per the criteria proposed by Lincoln and Guba of credibility, dependability, confirmability, and transferability [28]. In terms of credibility, our research team members were all well-trained. They were also experienced in working with informal caregivers of individuals with dementia. For dependability, a detailed study protocol was prepared before the study period. The study methodology was also reviewed by experienced researchers to ensure it was appropriate. Furthermore, during the analysis all team members were included in the discussion of the coding and analysis throughout the research project and consensus was reached in terms of understanding the codes. Confirmability was achieved via investigators triangulating the results including consensus decision making through collaborative discussions and participation of the team holding different opinions. Transferability was achieved via purposeful sampling, this was to ensure a wide selection of the sample (i.e., with domestic helper and without domestic helper). Data saturation was used as the end point for the study, and saturation was achieved through regular meetings following the interviews until no new information was collected to form new themes or describe existing themes further.

## Results

The age of the 29 participants were between 46 and 72 years (mean = 56.3, standard deviation = 6.5), with the majority being females (*n* = 23), Chinese (*n* = 26), and daughter-caregivers (*n* = 20). Around half of the caregivers had hired a domestic helper to support their daily caregiving activities (*n* = 15). Please refer to Table 1 for the socio-demographics of the participants.

**Table 1:** Socio-demographic characteristics of the study participants (*n* = 29)

	Mean	Standard deviation
Age	56.3	6.5
	Frequency	Percentage
Gender		
Male	6	21%
Female	23	79%
Ethnicity		
Chinese	26	90%
Malay	2	7%
Indian	1	3%
Relationship with the PWD		
Spouse	2	7%
Daughter	20	69%
Son	4	14%
Others (child-in-law or friend)	3	10%
Hired a domestic helper to support daily caregiving activities		
Yes	15	52%
No	14	48%

**Fig. 1:**
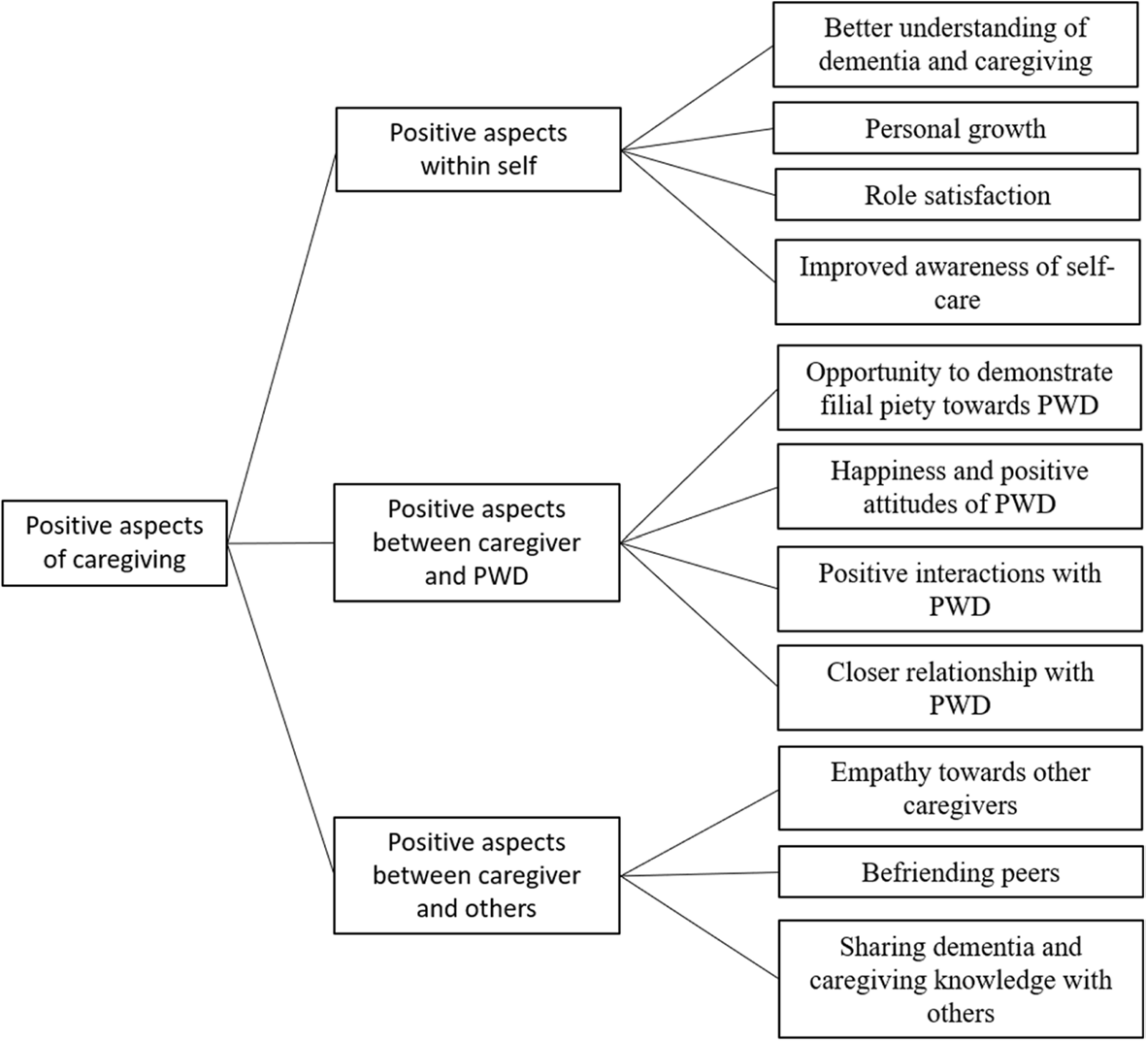
Themes identified pertaining to positive aspects of caregiving

Overall, three major themes with 11 sub-themes emerged on the positive aspects of caregiving among local informal caregivers of PWD. The three major themes were: (i) positive aspects within self, (ii) positive aspects between caregiver and PWD, and (iii) positive aspects between caregiver and others. Please refer to Fig. 1 for the details of the sub-themes under each major themes.

### Positive aspects within self

Taking care of PWD was also an opportunity for caregivers to learn about dementia and to improve their skills in caring for their loved ones. Such experiences can lead to personal growth such as becoming more resilient or more patient since they need to constantly adjust themselves to provide better care. Caregiving efforts may also lead to rewards among caregivers such as role satisfaction, especially when they are satisfied with their care provision or the care is appreciated by their significant others (including PWD). Lastly, as informal caregiving requires a lot of effort and dedication, it can also increase caregivers’ awareness on the importance of their own health. This is a prerequisite of good quality care provision as well.

#### Better understanding of dementia and caregiving

Providing care to PWD enabled caregivers to closely observe how individuals with dementia behaved daily. Simultaneously, they also needed to handle the challenges encountered during this process. Both provided opportunities for caregivers to learn about dementia and how to take care of PWD’s daily needs. In the current study, several caregivers reported that they understood dementia and caregiving better after taking up the caregiver’s role. Such knowledge was also helpful for caregivers when it was relevant to their work. For instance, one caregiver who was a traditional Chinese medicine physician talked about how taking care of the PWD helped him gain experience that could be helpful in his work in the future.

'I mean this is again the journey of learning. So, we discover that it’s actually not right because if you don’t engage her, she lost that communication skill, which in a way she eventually lost it… ‘– p04, daughter/51

‘…I think I’m wiser than some nurses in XXX hospital [hospital name] whose job is not looking after persons with Alzheimer’s full day. A person who is in ward A or B may not be Alzheimer’s right? But a person who can share profound informal caregiving experiences of Alzheimer’s with you would be the persons who is the main caregivers…’ – p06, son/68

‘I can gain experience you know… because I am a TCM (traditional Chinese medicine) physician, so it does help me to gain experience that in the future if I come across this type of dementia patient, I know how to deal with them, how to communicate with them, how to talk to them…’—p22, daughter/54

#### Personal growth

Other than knowledge gain, taking care of PWD also lead to personal growth as caregivers often had to adapt themselves to better handle the challenges encountered during this process. In the current study, some participants specifically mentioned that being an informal caregiver of PWD had changed them, such as becoming a stronger and more resilient person.

‘… if you ask me, I think I’m… now looking back… I really emerged as a stronger person. I mean in a more positive way. Actually, all these are really a learning journey for me, yeah. Everything I learned, everything I experienced, no matter how bad, how positive, to me I really gained everything out of it.’—p05, daughter/62

'…patience…I am an impatient person. Now I become very patient because no choice and when you face the circumstances, you have to do it…’ – p22, daughter/54

'…one thing is that caring for my parents has turned me in to a very caring person…in a way I am more capable on how to find resources…’—p25, daughter/47

#### Role satisfaction

One important indicator for caregivers to assess the success of their caregiving efforts was the status of the PWD they were caring for. Caregivers felt a stronger sense of satisfaction especially if they believed that their care was helpful to the PWD. In our study, multiple caregivers stated that they felt rewarded when the PWD’s condition (e.g., health and behaviours) stabilized or did not deteriorate as expected, with their care.

‘…when you see there’s an improvement… to you… it’s a great achievement … her body weight went down by 50% after my sister passed away[…] But now she is gaining back her weight… So, to me that’s an achievement…it’s a sign of …it’s an acknowledgement of something is right.’—p04, daughter/51

‘For what I think- partly why I think my mum is not deteriorating so badly is because we interact with her very much. Nonsense is also okay, to her is not nonsense. To us is nonsense, to her is something [referring to interaction with the PWD]. She’s happy, she’s satisfied…’ – p16, daughter/58

‘… it’s already 2 years and with the assessment with the clinic every 6 months, I’m seeing her … not deteriorating at the rate that the doctor has kind of …like you know tell me… it’s comforting seeing my mom under my care, … she’s still on the physical part … that is very, important … So I’m happy, I’m still positive that I’m still seeing her well enough as in not have to go through all that, yeah…’—p18, daughter/46

#### Improved awareness of self-care

Several participants stated that taking care of PWD raised their awareness of self-care. Some believed that they should take care of themselves first before caring for PWD.

‘I realized if you don’t take care of yourself, it’s very hard to take care of your [care recipient] … I realized social connections are important. It’s very easy to isolate yourself especially when you are very down … but I force myself out to … like this talking to you, it also helps.’—p09, daughter/53

‘… I need to be strong. Besides that, I also have my own health challenges so I need to help myself first before I can help her. If I’m not strong, how can I help her right?’ – p24, daughter/66

In other scenarios, caregivers may also develop their awareness on self-care in face of adversities during this process or through the interaction with healthcare professionals.

‘But self-care is still something I’m learning. Because before my burnout, I don’t know what is self-care. I really don’t know, it’s like …uh…so after going through the caregiver burnout, my depression episodes and stuff like that, then I realized that self-care is … it should be part of my life actually …integrated in my life, yah…’—p25, daughter/47

‘I mean it makes me more aware that this [dementia] runs in my family, so the doctor had already told me to be mentally prepared that it will happen to me and things that I should do to kind of try to delay that for as long as possible, like … to keep mentally active, physically active, socially active. So those are things that I’ve taken on board for myself as well…’—p26, daughter/49

### Positive aspects between caregiver and PWD

Other than the positive aspects within caregivers themselves, taking care of PWD can also enhance the relationships between caregiver and PWD, which in turn can lead to positive aspects between them, such as the chance to demonstrate filial piety, observing the happy moments of PWD, positive interaction with PWD and improved relationship with PWD.

#### Chances to demonstrate filial piety towards PWD

In the Asian-context, informal caregivers often viewed caregiving as an opportunity to demonstrate filial piety towards the PWD. With this opportunity they could give back the love they had received to PWD. In this study, quite some caregivers endorsed such a reciprocity notion underlying filial piety.

‘… because it’s intimate and because you are doing it for your father and your mother, you speak with a lot more poignancy right, a lot more meaning because it is your blood, it is your family, and you speak with a lot more passion and sincerity. Not from a book, I’m not reading out from a book you now. It is my own… mind and body.’ – p06, son/68

‘… the nice thing is you have… I mean it’s your mother and it’s a privilege I guess, you have to see it that way, I can have the time to spend with her you know. I mean as the years go, she’ll probably fade off even more. As it is now, on and off she can’t figure out, she knows I am her daughter but she has not said my name in a long time.’—p09, daughter/53

‘At least I have the chance to take care of her because last time she always takes care of me and then now at least I have the chance to take care of her.’ – p22, daughter/54

A few caregivers also talked about the unconditional love towards the PWD, and this was different from the reciprocity which is mentioned above. Instead, it is more of a responsibility, regardless of their earlier relationship with the PWD. This is from a traditional philosophy based on filial piety.

‘…the positive thing is […] I think the basis of us all is your intrinsic happiness, it takes you far and can sustain. What is that intrinsic happiness is because like for my mother it’s the unconditional love. Only that unconditional love can take you far regardless of how challenging the situation…’—p05, daughter/62

‘…I still feel very blessed that she’s with me and I have an understanding husband you know, so that I’m able to take care of her, that I’m still fit to take care of her, yeah […] it’s really the unconditional love when you come to taking care of [person with] dementia. It’s really unconditional. It’s one way giving you know, one-sided.’—p18, daughter/46

#### Happiness and positive attitudes of PWD

Happiness and positive attitudes of PWD were reported as positive aspects of caregiving according to many caregivers in the current study. Observing the happy moments of PWD also sparked positive feelings within the caregivers. In the current study, several caregivers stated that ‘we would be happy if she (PWD) is happy’.

'Positive experiences? Yah, at times they are very happy. Yeah…I mean…that also makes your day. Because like sometimes you are watching cooking program, then they are also happy, they will say that last time I can make this… I can make that …I’m so good in all these you see…they will be in a very happy, cheerful mood. So, we are also happy.’—p03, daughter/56

‘…when you see her smile, you know like when she’s happy with certain things, I mean to me every smile is worth a lot … it’s like a… reward to see her happy […] so you see that her behaviour, she becomes happier. She becomes calmer and she is eating better…this is her weight gain… to me that is the reward…’ – p04, daughter/51

‘… she still has that very positive outlook that I can go out with her […] she’s still fun to go with, if she has any interesting things she’ll still talk about. I mean it’s still that positive interaction. Whatever minimal it may be, comparatively to what she was in the past […] and she’s very cheery in the sense you look at her she smiles that kind of thing…’—p10, daughter/56

#### Positive interactions with PWD

At times, caregivers had positive interactions with PWD, and these interactions lead to their emotional fulfillment and happiness. This point was endorsed by the majority of the study participants. Furthermore, one caregiver also mentioned that these positive interactions, emotional fulfillment and happiness could recharge him and enable him to sustain his caregiving efforts.

‘… my mum has this very funny gesture. She always says that in her words, in our dialect, “you are the best… you are the best among…to look after me” She knows! She knows it’s me, so she said “you are the best, you are the best” And she kept saying that. And when you see her smile, you know like when she’s happy with certain things…’—p04, daughter/51

‘The one positive experience recently was, which was so wonderful was suddenly she looked at me and I think at that point, all the things, all the switches were on and she looked at me and she told me, ‘XX [caregiver name], thank you very much you saved my life’. So what more can I ask for?’ – p20, son-in-law/64

#### Closer relationship with PWD

A few caregivers felt that providing care to the PWD enabled them to know the PWD better and this ultimately improved their relationships with PWD.

' I would say we’re more, how to say, because she’s, last time she’s independent you know, she will go out with her friends and all that so we really don’t, I would say we’re closer.’ -p11, daughter/54

‘You know in Chinese family, we don’t really touch-touch, because we don’t… we just care for, I mean the way that I know how to do it is to care for her. And then she shows her care by like sometimes like she will give me money, $10, $20 you know, thank me for helping her. For me, before that she never, she doesn’t… yeah, in a sense like she looks at me kindly … now. Last time is like very critical on me, yah.’—p21, daughter/60

‘…with my mother, sometimes she can be very cute, very childish. So I really enjoy those moments. She will tell me about her past, how she grows up…I learned so much about her…’—p24, daughter/66

### Positive aspects between caregivers and others

Taking care of PWD might also lead to positive aspects towards others including empathy towards other caregivers, make new friends throughout the journey, and being able to share knowledge on dementia and informal caregiving with friends which might help others.

#### Empathy towards other caregivers

A few participants reported that taking care of the PWD helped them to understand how tough it is being a caregiver, as such they were able to understand other caregivers’ difficulties and show more empathy towards them.

‘…you see people taking care of the elderly, absolute respect because you know they probably… face the same situation or worse… there are people taking care of those children with … cerebral palsy… then we tell her maybe I’m taking care of my mom for probably 10 years but they’re going to take care of this one for maybe 20–30 years that kind of thing, then you feel sorry for them. Relatively you feel that you’re better…’—p08, son/56

‘In a way we are very lucky because we could afford it but having said that then we imagine what about other people who are going through the same and who don’t have this means, the kind of horrible additional financial burden is quite scary.’ – p20, son-in-law/64

#### Befriending peers

A few caregivers stated that they had made good friends with their peers along the caregiving journey.

' The friends I made along the way have also been a help, those from the support group and all that…’—p09, daughter/53

‘… so these are very positive, and of course making good friends, getting to know other caregivers through my journey as a caregiver, I know of other similar like-minded people who are genuine in my care journey…so things like that.’ – p25, daughter/47

#### Sharing dementia and caregiving knowledge with others

Better understanding of dementia and caregiving could bring other benefits, for instance, some caregivers suggested that they were able to share their knowledge and experiences with others (e.g., friends and peers), and such sharing helped others a lot.

‘For me the good thing is… there’s privilege of taking care of my mom, also the ability to, the lessons that we learned … So … if there’s somebody else that comes along with the same issue we can share openly. Like right now I have friends calling me. A lot of us are in that same stage you know, so it’s a great help.’—p09, daughter/53

‘… if my friends have certain things that they will ask me ‘what can I do’, or ‘what’s the step I should take the process?’ I will be able to give them some of my information. OK…like how, what to do when you go to clinic or hospital visit stuffs like that…’ – p25, daughter/47

## Discussion

Taking care of PWD is very stressful and full of challenges. However, like two sides of a coin, this experience also gives caregivers opportunities for their personal growth and can lead to other gains. Our study has explored these positive aspects among informal caregivers of PWD in Singapore and identified three major themes. The first major theme was positive aspects within self. To caregivers, providing care to the PWD was also an opportunity to learn about dementia and caregiving, to improve their caregiving skills in order to provide better care, and to gain a sense of satisfaction after successfully executing the caregiver’s role and duties. Similar positive aspects on ‘better knowledge on dementia and caregiving’ and ‘role satisfaction’ were reported among previous studies of western [29–31] as well as Asian caregivers [19, 24]. As such, they seem to be universal positive aspects of caregiving which do not differ much across cultures. On the other hand, our study also identified a more unique positive aspect of caregiving of ‘improved awareness of self-care’. More specifically, our participants stated that they must take care of themselves first before providing good care to PWD. Although this particular positive aspect was not reported in previous qualitative studies [18], this theme echoes with findings from quantitative research on the relationship between caregivers’ health and the quality of care received by PWD. For instance, poorer mental health of caregivers was reported to be associated with earlier institutionalization of PWD [32, 33] and higher mortality of PWD [34].

The second major theme was positive aspects between caregivers and PWD. Taking care of PWD enabled caregivers to demonstrate filial piety towards PWD. A similar positive aspect of reciprocity was reported among a few other studies conducted among western caregivers [18]. But there are some differences between these two concepts. Reciprocity is about giving back love [18] and it usually requires a prerequisite of positive relationship with the PWD before caregiving [30, 35]. In comparison, filial piety is about respecting and servicing family members especially parents [36]; and it is more like a social norm and reflects a sense of duty in which reciprocity is one major factor but it also includes other aspects such as unconditional material and emotional support of elderly parents [37, 38]. Moreover, filial piety among our participants also entailed the theme of ‘happiness and positive attitudes of PWD’ with quotes such as ‘we are happy if the PWD is happy’. This positive aspect was not reported in the previous studies conducted in western countries, indicating that it might be specific to the Asian context. There were two other positive aspects of caregiving under this theme, namely ‘positive interaction with PWD’ and ‘closer relationships with PWD’. These two positive aspects, although named differently such as emotional rewards or relationship gains in other studies, have both been reported elsewhere [18].

The last major theme was positive aspects between caregivers and others. The first positive aspect under this theme was improved empathy towards other caregivers. Empathy is the ability to put yourself in someone else’s shoes [39]. Providing care to the PWD enabled caregivers to really understand the challenges of caregiving, as such they would be more likely to respond to the emotional states of other caregivers through improved empathetic accuracy (i.e., a more accurate understanding of the situation) [40, 41]. Another unique theme is ‘befriending peers’. Although research has highlighted that caregiving can lead to relationship gains, however, the focus was mainly on the relationships with PWD or other family members [18, 19]. Our study suggested that caregivers may also make good friends with their peers and support each other along the way. The last sub-theme under this theme was about ‘sharing dementia and caregiving knowledge with others’. A similar theme was reported among a group of Hong Kong caregivers, and that study also suggested that caregivers might gain a sense of usefulness from such sharing [24].

Findings from our study have several practical implications. Firstly, we had used the Positive Aspects of Caregiving scale developed by Tarlow et al. [42] in a previous study to explore positive aspects of caregiving among local caregivers quantitatively, and found that this scale only had an acceptable fit [43]. Based on our conversations with the participants (as the data were collected via interviewer-administered questionnaire), we felt that some important components of positive aspects of caregiving among local caregivers may not have been captured by that scale. With the current qualitative study, we can infer that the missing components could be ‘awareness of self-care’, ‘chances to demonstrate filial piety’ and all aspects of interpersonal positive aspects of caregiving. As such, future studies might consider developing new instruments which include these new themes to measure positive aspects of caregiving locally and among other Asian informal caregivers, and test if it might yield a better fit. Secondly, studies had demonstrated that positive aspects of caregiving could buffer the negative consequences of caregiving [11, 14] and it was associated with lower depressive symptoms and burden among the caregivers [44]. Thus, the ability to identify positive aspects of caregiving seems beneficial to caregivers. Clinicians should consider focusing on the positive experiences encountered by caregivers in the clinical settings to improve the mental well-being of informal caregivers. Lastly, some researchers have started to use benefit-finding interventions to improve positive aspects of caregiving among informal caregivers [45, 46] and the preliminary evidence so far is supportive [47, 48]. To better support future research on benefit-finding intervention locally and in Asia, a list of positive aspects of caregiving within the Asian context could be helpful in the sense that it can guide the caregivers to reflect positively. Once caregivers are used to this thinking style, they can then practice by themselves and identify more positive aspects to further buffer their own stressors.

This study has several strengths. Firstly, we have adopted a variety of strategies to increase the rigor of the analysis, including a pre-prepared research protocol, the use of an interview guide, standardization of the data collection procedures, a multi-person coding team, consensus decision making and etc. These strategies have significantly increased the trustworthiness of the study findings. Secondly, the SRQR checklist [25] was used to guide the reporting, which will ultimately improve the transparency of this study. Nevertheless, it should also be viewed with the following limitations in mind. Firstly, the study sample was largely Chinese, with only a few Malays and Indians. It is important to include more caregivers of other ethnicities to understand if there are any ethnic differences. Nonetheless, our sample is comparable with the previous qualitative study in Singapore [19], and we believe that our findings are important to understand positive aspects of caregiving among informal dementia caregivers in Singapore as well as in the broader Asian context. Secondly, since convenience sampling was used and caregivers were also invited to refer their peers to join, there might be selection bias as the participants might be only from some relatively smaller caregiver circles.

## Conclusion

This study explored positive aspects of caregiving among informal caregivers of PWD in Singapore through a qualitative approach. A total of three major themes with 11 sub-themes were identified. The three major themes identified were positive aspects within self, positive aspects between caregiver and PWD, and positive aspects between caregiver and others. Apart from several universal positive aspects of caregiving themes such as ‘personal growth’ or ‘closer relationship with PWD’, our study identified a few unique-to-Asia themes like ‘improved awareness of self-care’ and ‘chances to demonstrate filial piety’. Thus, it may be necessary to develop more localized instruments to measure positive aspects of caregiving among informal caregivers of PWD in Asia. For future studies it would be helpful to cultivate caregivers’ ability to identify positive aspects of caregiving, and it is also necessary to take these cultural-specific positive aspects of caregiving into consideration especially in the Asian context.

## Supplementary Information

**Additional file 1. **

## Data Availability

All individual data from this study resides with the Office of Research, Institute of Mental Health. Data is not available for online access, however, readers who wish to gain access to the data can either write to the corresponding author or to the Clinical Research Committee, Institute of Mental Health/Woodbridge Hospital Secretariat at IMHRESEARCH@imh.com.sg. Access can be granted subject to the Institutional Review Board (IRB) and the research collaborative agreement guidelines. This is a requirement mandated for this research study by our IRB and funders.

## Abbreviations

PWD: Persons with dementia
SRQ: Standards for Reporting Qualitative Research
TCM: Traditional Chinese medicine
