# Supplementary material for: Positive aspects of caregiving among informal caregivers of persons with dementia in the Asian context: a qualitative study
Source: BMC Geriatr. 2023 Jan 27;23:51. doi: 10.1186/s12877-023-03767-8 (PMC9883086; doi:10.1186/s12877-023-03767-8)
Supplement: Supplementary file 1 — Additional file 1. [file 12877_2023_3767_MOESM1_ESM.docx]

Supplementary table

| Meaning Units | Code | Subtheme | Major theme |
| --- | --- | --- | --- |
| … that was how I managed to keep it up, because of the fear. I need to look after my family, I look after myself - p01 | Reasons for self-care | Improved awareness of self-care | Positive aspects within self |
| I realize social connections are important… - p09 | Means of self-care |  |  |
| If I’m not strong, how can I help her right? – p24 | Importance of self-care |  |  |
